# Draft Genome Sequence of the Plant-Pathogenic Soil Fungus *Rhizoctonia solani* Anastomosis Group 3 Strain Rhs1AP

**DOI:** 10.1128/genomeA.01072-14

**Published:** 2014-10-30

**Authors:** Marc A. Cubeta, Elizabeth Thomas, Ralph A. Dean, Suha Jabaji, Stephen M. Neate, Stellos Tavantzis, Takeshi Toda, Rytas Vilgalys, Narayanaswamy Bharathan, Natalie Fedorova-Abrams, Suman B. Pakala, Suchitra M. Pakala, Nikhat Zafar, Vinita Joardar, Liliana Losada, William C. Nierman

**Affiliations:** a Department of Plant Pathology, North Carolina State University, Raleigh, North Carolina, USA; b Department of Plant Science, McGill University, Quebec, Canada; c Leslie Research Centre, Queensland, Australia; d Department of Biological Sciences, University of Maine, Orono, Maine, USA; e Bioresource Sciences, Akita Prefectural University, Akita, Japan; f Department of Biology, Duke University, Durham, North Carolina, USA; g Department of Biology, Indiana University of Pennsylvania, Indiana, Pennsylvania, USA; h J. Craig Venter Institute, Rockville, Maryland, USA

## Abstract

The soil fungus *Rhizoctonia solani* is a pathogen of agricultural crops. Here, we report on the 51,705,945 bp draft consensus genome sequence of *R. solani* strain Rhs1AP. A comprehensive understanding of the heterokaryotic genome complexity and organization of *R. solani* may provide insight into the plant disease ecology and adaptive behavior of the fungus.

## GENOME ANNOUNCEMENT

The soil fungus *Rhizoctonia solani* anastomosis group three (AG-3) is an economically important pathogen of eggplant, pepper, potato, and tomato. The fungus can degrade organic matter in soil as a saprobe and represents an important evolutionary link between beneficial and plant disease-causing fungi (1). The fungus exists in nature as a heterokaryon, with at least two genetically distinct nuclear genomes per cell (2). In this study, a field isolate of *R. solani* AG-3, strain Rhs1AP (ATCC MYA-4579) sampled from an infected potato stem in Maine in 1988 was sequenced using Sanger (4-, 10-, and 40-kb insert sizes) and GS-FLX 454 (fragment and 20-kb mate pair) technologies. The genome was assembled using the Celera Assembler 5.1 and CLC *de novo* assembler. The draft genome of *R. solani* strain Rhs1AP is 51,705,945 bp and consists of 326 scaffolds containing 6,040 contigs over 360 bp, with a contig *N*_50_ value of 25,869. The expected total size of the heterokaryotic genome of Rhs1AP was approximately 86 Mb, based on an optical map of the chromosomes (data not shown), suggesting that some Rhs1AP contigs collapsed during the assembly due to their high similarities. A read coverage analysis showed that the majority of the contigs (36.9 Mb) had similar coverages (34×), while 6.7% (2.7 Mb) and 6% (2.3 Mb) of the contigs were covered at 17× and ≥51×, respectively. These results suggest that contigs with 34× coverage are present twice as duplicated copies in the genome, while those with 17× and 51× are present once or three times, which added up to 83 Mb approximating the expected genome size. The genome of strain Rhs1AP was annotated using the eukaryotic annotation pipeline of the J. Craig Venter Institute (JCVI) and has 12,726 predicted genes. The Rhs1AP genome posed many inherent assembly challenges, since the fungus has multinucleate heterokaryotic hyphal cells and is an aneuploid, with a highly repetitive genome that resulted in relatively fragmented assemblies. Although most fungal biologists who study filamentous fungi in the *Agaricomycetes* (i.e., the mushroom forming fungi and their allies) have chosen to sequence the haploid component of a dikaryotic or heterokaryotic strain, this is not the predominant life stage of these fungi in nature. This experimental approach reduces the challenges associated with the assembly and annotation of the genome sequence data, but it provides only a partial understanding of its genome organization. The annotated draft genome sequence of *R. solani* strain Rhs1AP will provide a foundation of knowledge for understanding the relative contributions of genome organization and nuclear heterogeneity to the adaptive behavior, survival, and plant disease ecology of the fungus.

### Nucleotide sequence accession number.

The whole-genome shotgun project for Rhs1AP has been deposited at DDBJ/EMBL/GenBank under the accession no. JATN00000000.
